# Case-control Studies on the Relationship between Onchocerciasis and Epilepsy: Systematic Review and Meta-analysis

**DOI:** 10.1371/journal.pntd.0002147

**Published:** 2013-03-28

**Authors:** Christoph Kaiser, Sébastien D. S. Pion, Michel Boussinesq

**Affiliations:** 1 Basic Health Services Kabarole & Bundibugyo Districts, Fort Portal, Uganda; 2 Institut de Recherche pour le Développement, UMI 233, Montpellier Cedex 5, France; Imperial College London, School of Public Health, United Kingdom

## Abstract

**Objective:**

A systematic review and meta-analysis of all available case-control studies on the relationship between onchocerciasis and epilepsy. Because age and level of onchocerciasis endemicity in the area of residence are major determinants for infection, an additional analysis was performed, restricted to studies achieving control of these confounding factors.

**Data sources:**

Medical databases, the “African Neurology Database, Institute of Neuroepidemiology and Tropical Neurology, Limoges,” reference lists of relevant articles, commercial search engines, up to May 2012.

**Methods:**

We searched for studies examining infection status with *Onchocerca volvulus* in persons with epilepsy (PWE) and without epilepsy (PWOE) providing data suitable for the calculation of pooled odds ratios (OR_p_) and/or standardized mean differences (SMD) using random-effects models.

**Results:**

Eleven studies providing data of qualitative skin biopsies for diagnosis of onchocerciasis were identified. Combined analysis on the total sample of 876 PWE and 4712 PWOE resulted in an OR_p_ of 2.49 (95% confidence interval (95%CI): 1.61–3.86, p<0.001). When this analysis was restricted to those studies achieving control for age, residence and sex (367 PWE, 624 PWOE), an OR_p_ of 1.29 (95% CI: 0.93–1.79; p = 0.139) was found. Presence of nodules for diagnosis of onchocerciasis was analyzed in four studies (225 PWE, 189 PWOE; OR_p_ 1.74; 95%CI: 0.94–3.20; p<0.076), including two studies of the restricted analysis (106 PWE, 106 PWOE; OR_p_ 2.81; 95%CI: 1.57–5.00; p<0.001). One study examined quantitative microfilariae counts in patients without preceding microfilaricidal treatment and demonstrated significantly higher counts in PWE than in PWOE.

**Interpretation:**

Our results strengthen the hypothesis that, in onchocerciasis foci, epilepsy and infection with *O. volvulus* are associated. Analysis of indicators giving information on infection intensity, namely nodule palpation and quantitative microfilaria count in untreated patients, support the hypothesis that intensity of infection with *O. volvulus* is involved in the etiology of epilepsy.

## Author Summary

Onchocerciasis is known as a cause of skin and eye disease infecting a great number of people, mainly in rural Africa. In the endemic areas, infection status and severity is essentially determined by the duration and intensity of exposure to the parasite and to host sex. A link between onchocerciasis and epilepsy has been suggested over a long time but the existence of a definite association is still controversial. Based on a comprehensive literature review of case-control studies on this issue, the authors found a significant association between epilepsy and a positive infection status with *Onchocerca volvulus*. This relationship was less pronounced when the analysis was restricted to those studies giving evidence of sufficient control for age, endemicity level of the area of residence and sex. When within this restricted sample indicators giving information on infection intensity, namely nodule palpation and microfilarial density in untreated patients, were taken into consideration, again a significant result was found. In their overall constellation these results corroborate the hypothesis that epilepsy in endemic areas is caused by a disease process induced by infection with *O. volvulus*, especially in patients with severe infection.

## Introduction

Onchocerciasis is a parasitic disease caused by infection with the nematode worm *Onchocerca volvulus*. Infective larvae are transmitted through bites of flies of the genus *Simulium* which breed along fast-flowing rivers throughout tropical Africa and some areas of Latin America and Yemen [1]. Whereas children in the first years of life are only exceptionally infected, the prevalence rises to virtually 100% in adults living in highly endemic areas, with usually a higher proportion in the male population [2]–[4]. Within one endemic area, the risk of infection may vary significantly over short distances even between neighbouring villages. Thus, the main determinants of infection status with *O. volvulus* are:

- Intensity of exposure: In communities living in villages exposed to onchocerciasis, prevalence and intensity of infection increase with the proximity to the breeding places and thus the frequency of the bites of vector flies and the number of infective larvae transmitted per bite [5]. Place of residence, usually specified as village of residence, is therefore considered a valid indicator of intensity of exposure.
- Time (duration) of exposure: For those born in an *O. volvulus* endemic area, this coincides with the age of the inhabitants. Especially in non-stable populations the time point of immigration has to be taken into account.
- Sex: Throughout all ages, males are more frequently infected than females in most endemic areas.

Following infection of the human host the parasite develops into adult worms of considerable size (length up to 50 cm for female worms) and aggregates within nodules which can be located in the sub-cutaneous layer of the skin but also in internal parts of the body [6]. Subcutaneous nodules can be used for diagnosis of onchocerciasis although this is of limited sensitivity because deeply seated nodules cannot be located by palpation [7]. Adult parasites can produce an estimate of 1300–1900 larvae (microfilaria, mf) per day [6] which invade the dermis of the host to be newly incorporated with the blood meal of the vector fly. The detection of mf in skin biopsies (skin snips) allows a more sensitive and specific diagnosis of *O. volvulus* infection compared to nodule palpation. Beyond that, the count of mf per mg of skin or per skin snip of standardized weight is also a measure of infection intensity [8], [9]. Mf were identified as the pathogenic stage of the parasite, and mf density was shown to be linked with the known disease manifestations of onchocerciasis in the skin and the eye.

An association between onchocerciasis and epilepsy was first suspected in the 1930's and 1940's in Latin American endemic areas [10]–[12] and there were sporadic anecdotal accounts mentioning a clustering of epilepsy in several African onchocerciasis foci as well [13]–[15]. Only with the implementation of community-wide treatment campaigns with the microfilaricidal drug ivermectin, which has been made available at no cost by its manufacturer since 1987 (Merck & Co., Whitehouse Station, NJ, U.S.A.), could this issue be investigated more systematically. Ecological studies carried out in onchocerciasis foci throughout West, Central and East Africa found a strong positive exponential relationship between the prevalence of onchocerciasis and that of epilepsy [16]. Differing from this association demonstrated at the community level, case-controlled studies on the issue gave equivocal results. A meta-analysis comparing onchocerciasis status in persons with and without epilepsy yielded widely deviating findings ranging from a relative risk (RR) of 6.80 (95% CL 3.00–15.20) suggesting a strong positive association to a RR of 0.84 (95% CL 0.74–0.95) suggesting a negative association [17]. When looked at more closely, the most extreme results included in this review originated from two studies carried out in the same endemic area in Western Uganda with a time interval of only one year [18], [19]. The discrepancy between the results of these studies was most likely due to shortcomings in study design and the selection of comparison groups [20].

Keeping in mind the strong dependency of onchocerciasis infection on age, *O. volvulus* endemicity in the area of residence, and sex, it is essential that comparative studies ensure that these major confounders are controlled if valid conclusions on a relationship between the parasite and disease manifestations are to be made. The present article provides a review of all case-control studies investigating the relationship between onchocerciasis and epilepsy carried out to date, with a focus as to whether age, residence and sex as the major determinants for infection status were controlled.

## Methods

Data for this article were entirely assessed from previously published work and no information that could identify individual patients is provided. Therefore, written consent and institutional ethical review are not required for this review. The PRISMA checklist and flow diagram [21] are available as supporting information (Diagram S1, Checklist S1).

Several medical databases (MEDLINE, ScienceDirect, Scopus) and the “African Neurology Database of the Institute of Neuroepidemiology and Tropical Neurology of the University of Limoges” (http://www-ient.unilim.fr/) were screened by use of the key words “onchocerciasis” AND “epilepsy”. Other sources, such as commercial search engines or unpublished congress proceedings, were searched with no specified limits, and reference lists of retrieved articles and reviews were screened for further records of relevance (Latest search: May 23, 2012). Full text of articles, theses and abstracts thus identified were independently examined by two of the authors (CK and MB) and eligibility for inclusion into the review was agreed upon by consensus. A study was considered eligible if it made a comparison of *O. volvulus* infection status in a group of people with epilepsy (PWE) to that in a comparison group of people without epilepsy (PWOE), and if it reported data allowing for the calculation of an odds ratio (OR) with *O. volvulus* infection status considered as exposure and epilepsy as outcome and its 95% confidence intervals (95% CI). The pooled OR was calculated as an overall measure of a connection between onchocerciasis and epilepsy in all studies providing compatible data.

In a second step, the analysis was restricted to those studies fulfilling defined criteria concerning their composition of age (time of exposure), residence (intensity of exposure) and sex (Age: Matching on age with a maximum interval of 5 years, *or* Standardization on age with a maximum interval of 5 years; Residence: Matching by village, *or* Stratification by endemicity level; Sex: Matching on sex, *or* Results reported separately for males and females). These criteria were based on the known relationship between the respective parameters and infection status with *O. volvulus* and were also guided by the suggestions made for epidemiological studies on the association between cysticercosis and epilepsy [22]. Those studies which met the pre-defined selection criteria were further classified according to the methods used for the ascertainment of *O. volvulus* infection: (i) Qualitative and/or quantitative detection of mf of *O. volvulus* in the skin, or (ii) Presence of subcutaneous nodules and/or number of nodules detected by palpation. Pooled ORs and 95% CI were calculated for studies presenting qualitative indicators of onchocerciasis infection whereas pooled standardized mean differences (SMD) and Cohen's d statistics were computed for studies presenting quantitative assessment of onchocerciasis infection [23]. All pooled calculations included a test of homogeneity of means. Pooled ORs were estimated using random-effects models (DerSimonian-Laird method) and heterogeneity of studies was assessed using Cochrane's Q chi-squared tests and the I^2^ statistics [24], [25]. The latter describes the percentage of total variation across studies that is due to heterogeneity rather than chance. Analyses were performed using the *metan* procedure [26] available in the Stata software (StataCorp, TX).

In order to discuss factors that might have influenced the results, information on the characteristics of each study site was also collected, and in some cases, the authors were directly contacted to get data not available in the publications. The levels of endemicity were defined using either the prevalence of microfilaridermia [27] or the prevalence of nodules [28], following whichever data were available.

## Results

### Result of the literature search

An overall number of 290 entries were found in the medical databases by use of the keywords “onchocerciasis” AND “epilepsy”, corresponding to a number of 210 distinct records after duplicates were removed. Out of these records, 16 articles, medical theses and abstracts representing 11 studies were found to include a control group for the investigation of a relationship between onchocerciasis and epilepsy [18], [20], [29]–[42]. Two records, each representing an additional eligible study [43], [44], and one unpublished congress abstract [45] containing relevant information about one of the above-mentioned studies were identified in other sources.

### Results including all identified studies

Information about all identified studies, including their design, the levels of endemicity for onchocerciasis in each site, and the onchocerciasis control activities conducted before the study is given in Tables 1 and 2. The article by König et al. [36] and the abstract by Schmutzhard et al. [45] from the Morogoro focus in Tanzania reported information on *O. volvulus* infection status of PWE and controls only as pooled data, based on skin snip microscopy combined with the results of a polymerase chain reaction (PCR) method searching for *O. volvulus* specific DNA in the skin. However, data on the prevalence of skin mf alone were provided in an abstract about the same study [37]. In their survey reported from Burundi, Newell et al. [38] used a skin scarification technique for the detection of *O. volvulus* mf which is considered equivalent to the standard skin snip procedure established by the Onchocerciasis Control Programme in West Africa (OCP). This was complemented by a serology assay in those with a negative scarification result and figures are given in the article on the proportions of subjects found positive either with the scarification method alone, or with both techniques [38]. In the study conducted by Kipp et al. [18], infection with *O. volvulus* was defined by the presence of skin mf or the presence of nodules. As the proportion of individuals harboring nodules but no mf in the skin is usually very low [46], we have considered that the prevalence given in this publication corresponded to the prevalence of skin mf. Thus, with the exception of two studies where onchocerciasis status has been evaluated by searching the presence of nodules only [40], or by the detection of circulating antigens only [43], all studies communicated data of qualitative skin biopsies allowing for the calculation of a pooled OR of 2.49 (95%CI: 1.61–3.86, p<0.001) (Table 2; Figure S1). Among these studies, heterogeneity in ORs was significant (Cochrane's Q test: p<0.001) and yielded an I^2^ value of 65.7%. Data on the prevalence of nodules in PWE and PWOE was provided in four studies [20], [39], [40], [44] from which we calculated a pooled OR of 1.74 (95%CI: 0.94–3.20, p = 0.076) (Table 2). Between-studies heterogeneity was not significant (p = 0.093) and I^2^ was assessed as 53.3%.

**Table 1:** Design of all eligible studies on the relationship between onchocerciasis and epilepsy by use of a comparison group.

Year of study, Country [Reference]	Identification/selection of participants		Assessment of diagnosis		No. of Persons included		Control for confounding factors		
	Persons with epilepsy (PWE)	Persons without epilepsy (PWOE)	Epilepsy^a^	Onchocerciasis^b^	PWE	PWOE	IoE^c^	ToE^c^	Sex
1991, Cameroon [29], [39]	Lists made by community leaders+ITW^a^ at onchocerciasis survey	Inhabitants without history of seizures identified in parasitological survey	Non-standardized ITW performed by MD	Nod q, Nod Q, mf q, mf Q	72	72	**+**	**+**	**+**
1993, Uganda [18]	ITW^a^ with household members from lists of local authorities	Household members without history of seizures	Non-standardized ITW performed by MD	Nod q+mf q	39	946	**+**	**−**	**−**
1994, Burundi [38]	Lists produced by local authorities and health center staff	Relatives of PWE from the same household	Non-standardized ITW performed by MD	mf q, serology	110	82	**+**	**−**	**−**
1994, Uganda [20]	PWE aged 10–19 years identified in a population-wide survey	Inhabitants without seizures identified in parasitological survey, age 10–23 y	SITW and NE performed by MD	Nod q, Nod Q, mf q	38	38	**+**	**+**	**+**
1995, Benin [32]	Random household sample, Door-to-door survey	Household members without history of seizures	Non-standardized ITW and NE by neurologist	mf q	13	517	**−**	**−**	**+**
1996, Burkina Faso [33]–[35] d	Population-wide survey, ITW^a^ of inhabitants aged >14 years	All inhabitants aged >14 years without history of seizures	SITW performed by health personnel	mf q	34	2006	**+**	**−**	**−**
1996, CAR [30]	Identification of PWE >14 years by health personnel and village leaders	Inhabitants without neurologic illness, age >14 years	SITW and NE by neurologist	mf q, mf Q	187	374	**+**	**+**	**+**
1996, Tanzania [41]	Participants of oncho. survey with history of epileptic seizures	Participants of oncho. survey without seizure history	SITW performed by MD	mf q^e^	34	380	**+**	**−**	**+**
1998, Mali [31], [42]	Population-wide survey, ITW^a^ of inhabitants aged >7 years	Inhabitants without history of seizures aged >7 years	SITW and NE by neurologist	mf q^f^	70	140	**+**	**+**	**+**
2000, Cameroon [44]	Identification of PWE by community key persons, age <34 years	Inhabitants attending for other illness, age <34 years	SITW by MD	Nod q, mf q, mf Q	83	53	**−**	**+**	**+**
2004, Cameroon [40]	Door-to-door survey in one village, ITW^a^ of all members of households accepting to participate	Subjects without history of seizures selected at random from participating households	SITW by MD trained in neurology+review by neurologists	Nod q	18	36	**+**	**+**	**−**
2005, Tanzania [36], [37], [45]	PWE attending specific epilepsy clinic	Relatives of PWE without history of seizures	SITW and NE by neurologist	mf q, mf Q, skin PCR	196	104	**+**	**−**	**−**
2005, Cameroon [43]	Identification in one village by health authorities	Not specified	Not specified	Circulating antigens	54	98	**+**	**−**	**−**

a Assessment of Epilepsy: ITW = interview; MD = medical doctor; NE = neurologic examination; SITW = standardized interview;

b Assessment of Onchocerciasis: Nod q = examination for presence of nodule(s); Nod Q = assessment of number of nodules; mf q = microscopic examination for presence of microfilariae in skin biopsies (no count); mf Q = assessment of skin microfilarial density; Nod+mf q = assessment of presence of nodules OR of mf; PCR = detection of parasite DNA in skin biopsy by using polymerase chain reaction.

c Control for confounding factors: IoE = Intensity of exposure (residence); ToE = Time of exposure (age/duration of residence).

d The article by Kabore et al. [33] reports data from only 5 of the 12 villages for which data are given in reference [34] and [35].

e Patients without skin microfilariae but having experienced a reaction to ivermectin treatment within 1 year were also considered infected.

f Results of eye examinations were reported in this study but are not considered in the present analysis.

**Table 2:** Case-control studies on the onchocerciasis-epilepsy relationship: study areas characteristics and odds ratios (OR).

[Reference]^a^, year of study; country; study area	Pre-control onchocerciasis endemicity level^b^	Duration of onchocerciasis control before study (years)^c^	Onchocerciasis endemicity level at time of study^b^	OR mf (95% CI)^d^	OR Nod (95% CI)^d^
[29], [39] a 1991; Cameroon; Mbam & Kim and Mbam & Inoubou, 17 villages	Hyper (Pmf>69%)	0	Hyper (Pmf>69%)	4.17 (0.45–38.32)	2.82 (1.43–5.56)
[20] a 1994; Uganda; Kabarole district, 13 villages	7 villages hyper, 6 meso/hypo	3 CDTI	7 hyper (Pmf>60%), 6 meso/hypo	1.67 (0.61–4.57)	2.77 (0.92–8.33)
[30] a 1996; CAR; Ouham and Ouham-Pende divisions, dispensaries in 3 towns	Area meso/hyper	5 CDTI	Meso/hyper	1.17 (0.82–1.68)	NA
[31], [42] a 1998; Mali; Tyenfala and Baguineda sub-divisions, 18 villages	7 villages hypo, 11 meso/hyper	11 CDTI+VC for 4 years (1994–1997)	All hypo (Pmf 9% and 23%)	2.04 (0.40–10.40)	NA
[18] 1993; Uganda; Kabarole district, two villages	1 village hyper, 1 village hypo	2 CDTI	1 hyper (Pmf 63%), 1 hypo (Pmf 19%)	7.31 (3.19–16.73)	NA
[38] 1994; Burundi; Bururi province, Buyengero & Burambi divisions	Area meso/hyper	0	Meso/hyper	2.49 (1.38–4.50)	NA
[32] 1995; Benin; Dassa-Zoumé sub-division, one town and neighboring villages	Area meso/hyper	1 CDTI+VC for 7 years (1988–1995)	Meso (Pmf 47%)	2.56 (0.78–8.41)	NA
[33]–[35] 1996; Burkina; Bougouriba province, 12 villages	Area hyper	VC for 16 years (1975–1990)	Hypo (Pmf 13%)	0.84 (0.29–2.40)	NA
[41] 1996; Tanzania; Ruvuma region, Songea district, one village	Area hyper	0^e^	Hyper (Pmf 68%)	3.50 (1.21–10.17)	NA
[44] 2000; Cameroon; Sanaga maritime division, Logbikoy hospital	Area hyper	1 CDTI^e^	Hyper (Pmf>80%)	3.76 (1.31–10.74)	0.98 (0.55–1.75)
[40] 2004; Cameroon; Sanaga maritime division, one village	Area hyper	3 CDTI^e^	Hyper (PNod 62.5%)	NA	1.38 (0.42–4.51)^f^
[36], [37], [45] 2005; Tanzania; Morogoro region, Ulanga district, Mahenge hospital	Area meso/hyper	8 CDTI	Meso	3.77 (2.18–6.52)	NA
Pooled studies (random effects model)				2.49 (1.61–3.86)	1.74 (0.94–3.20)

a The first four studies are those achieving control for intensity and time of exposure and gender. In these studies people with epilepsy were matched for gender, age and place of residence to one or two people without epilepsy; in the study by Kaiser et al. (2011), 5 of 38 pairs were not matched for sex.

b Pmf = prevalence of skin microfilariae (mf) in subjects aged ≥5 years; PNod = prevalence of nodules in males aged ≥20 years; Hypo = hypoendemic (Pmf<35% or PNod<20%); Meso = mesoendemic (35%≤Pmf<60% or 20%≤PNod <40%); Hyper = hyperendemic (Pmf≥60% or PNod≥40%).

c CDTI = Community-Directed Treatment with Ivermectin; VC = Vector control.

d OR mf = Odds ratio for epilepsy in patients with skin mf; OR Nod = Odds ratio for epilepsy in patients with nodules; 95%CI = 95% Confidence interval; NA = not assessable.

e Passive ivermectin treatment had been organized in these areas before the implementation of the CDTIs organized by the African Program for Onchocerciasis Control (APOC).

f OR calculated on the number of persons examined (6 of the 36 controls were “missing” for nodule palpation). If all the missing controls had nodules, the OR would be 1.00 (0.31–3.19) and if none of them had nodules, the OR would be 1.96 (0.62–6.22).

When the results of the above mentioned study from the Morogoro focus in Tanzania were analyzed based on the results of both methods used for detection of *O. volvulus* (microscopy and PCR) instead of microscopy alone, the proportion of participants with a positive *O. volvulus* infection status was found to increase more in PWE than in PWOE, resulting in an OR of 4.36 (95%CI: 2.63–7.24) [36] instead of 3.77 (95%CI: 2.18–6.51) (Table 3) [37]. In the study conducted by Newell et al. from Burundi [38] the OR was found at 2.49 (95%CI: 1.38–4.50) when only the results of skin scarification were considered, instead of 2.09 (95%CI: 1.07–4.09) when findings of skin scarification and serology were combined for analysis (Table 3). The study of Tume et al. [43] from Cameroon, which was exclusively based on detection of circulating antigens of *O. volvulus*, found no significant difference between the seroprevalence in 441 PWE (17.7%) and 98 PWOE (20.4%) (Table 3).

**Table 3:** Results of studies utilizing supplementary methods for assessment of *O. volvulus* infection (serology, polymerase chain reaction, circulating antigen).

Country, Year of study [Reference]	Method of assessment	Persons with epilepsy (PWE)		Persons without epilepsy (PWOE)		Odds ratio (95% CI)	P value
1994, Burundi [38]		n = 110^a^		n = 82			
		Oncho +^b^	Oncho −b	Oncho +	Oncho −		
	Skin scarification alone	69	41	33	49	2.49 (1.38–4.50)	p = 0.001
	Skin scarification and Serology combined	90	20	56	26	2.09 (1.07–4.09)	p = 0.023
2005, Tanzania [36], [37], [45]		n = 200 (196)^a^^,^ c		n = 100 (104)^a^^,^ c			
		Oncho +	Oncho −	Oncho +	Oncho −		
	Skin snip alone	103	97	22	78	3.77 (2.18–6.52)	p<0.0001
	Skin snip and skin PCR combined	135	61	35	69	4.36 (2.62–7.24)	P<0.0001
2005, Cameroon [43]		n = 441^a^		n = 98^a^			
		Oncho +	Oncho −	Oncho +	Oncho −		
	Circulating antigens	78	363	15	83	1.19 (0.65–2.17)	p = 0.29

a n = number of persons examined.

b Oncho + = No. of persons with positive onchocerciasis infection status; Oncho − = No. of persons with negative onchocerciasis infection status.

c Numbers of persons examined differed slightly between reports from this study. Data for skin snip results alone are presented as given in reference [37], and for skin snip and skin PCR results combined as given in reference [36].

### Results from studies fulfilling pre-defined control criteria

Four studies from Cameroon [29], [39], Central African Republic (CAR) [30], Mali [31] and Uganda [20] were found to meet with the criteria for sufficient control of age, residence and sex (Table 2). All these studies applied a pair-matching protocol for age and residence, and all but one were also fully matched for sex. In one study from western Uganda an equal distribution of male and female participants was documented although 5 out of 38 pairs were of deviating sex [20]. The endemicity level of the different areas at the time of the realization of the studies varied considerably and was apparently related to the duration of treatment campaigns with ivermectin and, in one case, to previous vector control activities (Table 2). All studies included in the restricted analysis used comparable methods for the assessment of epilepsy diagnosis.

Qualitative analysis of skin biopsies (prevalence of infection) yielded a non-significant result for the individual studies as well as for all studies in combination (pooled OR: 1.29, 95% CI: 0.93–1.79; p = 0.139) (Table 4). No statistically significant heterogeneity was found between studies (p = 0.59; I^2^ = 0%). Two studies also reported the result of quantitative mf counts. When this was done in a cohort of patients in the CAR who had been receiving microfilaricidal treatment for five years, almost no difference was found between patients with epilepsy and controls [30]. In contrast, a highly significant result was obtained in a study from Cameroon performing quantitative mf counts in patients who had not yet received ivermectin at the time of the skin biopsy [29]. Pooling the two studies yielded a non significant standardized mean difference (SMD) (p = 0.407) and revealed a substantial heterogeneity between them (p<0.001; I^2^ = 92.1%) (Table 4).

**Table 4:** Case-control studies on the onchocerciasis-epilepsy relationship: results of studies controlling for confounders.

Method of assessment	Reference	Persons with epilepsy (PWE)		Persons without epilepsy (PWOE)		Odds ratio (95% CI)	SMD^a^	P value
Skin biopsy								
Qualitative		No. mf+^b^	No. mf−b	No. mf+	No. mf−			
	Druet-Cabanac et al. [30]	74	113	134	240	1.17 (0.82–1.68)		
	Farnarier et al. [31] c	3	67	3	137	2.05 (0.40–10.40)		
	Boussinesq et al. [29]	71	1	68	4	4.18 (0.45–38.32)		
	Kaiser et al. [20]	29	9	25	13	1.68 (0.61–4.57)		
	Pooled result (random effects)					1.29 (0.93–1.79)^d^		p = 0.139
Quantitative		Mean mf density (±SD)^a^		Mean mf density (±SD)^a^				
	Druet-Cabanac et al. [30]	26±42		28±48				n.r.^e^
	Boussinesq et al. [29]	288±274		141±173				<0.0001
	Pooled result (random effects)					0.28 (−0.39–0.96)	0.407^f^	
Nodule palpation								
Qualitative		No. Nod+^b^	No. Nod −b	No. Nod+	No. Nod −			
	Kaiser et al. [20]	11	23	5	29	2.77 (0.92–8.33)		0.065
	Pion et al. [39]	49	23	31	41	2.82 (1.43–5.56)		0.005
	Pooled result (random effects)					2.80 (1.57–5.00)^d^		<0.001
Quantitative		Mean No. of Nod (±SD)		Mean No. of Nod (±SD)				
	Kaiser et al. [20]	0.45±0.72		0.18±0.45				0.061
	Pion et al. [39]	1.14±1.04		0.82±1.30				0.187
	Pooled result (random effects)						0.384 (0.02–0.745)	0.037^f^

a SMD = Standardized Mean Difference; SD = Standard deviation.

b No. mf+/No. mf− = Number of persons with/without microfilaria in skin biopsy; No. Nod +/No. Nod − = Number of persons with/without palpable nodule.

c Results of eye examinations not considered.

d Mantel-Haenszel weighted summary Odds ratio.

e n.r. = not reported in original study [30].

f Test of Cohen's Standardized Mean Difference [23].

Nodule palpation was analyzed in the studies conducted in Uganda [20] and in Cameroon [39] and produced congruent results, with a significantly higher prevalence of nodule carriers (pooled OR: 2.80, 95%CI: 1.57–5.00; p<0.001) (Table 4), as well as higher nodule counts in patients with epilepsy (SMD: 0.384, 95%CI: 0.02–0.75; p = 0.037). No significant heterogeneity was found between the Ugandan and Cameroonian studies either for the prevalence or the number of nodules (p = 0.981 and p = 0.64, respectively; and I^2^ = 0% for both indicators of *O. volvulus* infection).

## Discussion

In a search for comparative studies on the relationship between onchocerciasis and epilepsy published until to date, we identified a total number of 13 studies of which 11 studies presented data of qualitative skin biopsies for the diagnosis of onchocerciasis amenable to combined analysis. In this sample of 876 epilepsy patients and 4712 control subjects we found a highly significant (2.5-fold, p<0.001) increase in the risk of having a positive skin biopsy for PWE if compared to PWOE. Some of these studies were probably subject to bias because of limitations in the study design or because they did not make sufficient use of measures to control for confounding factors. In one study [18], a substantial overrepresentation of control subjects with residence in low endemic areas probably led to an over-estimate of the reported positive association. Another study [33]–[35] was carried out in an area in Burkina Faso which at the time of the survey had been subject to effective vector control measures as part of the Onchocerciasis Control Programme in West Africa (OCP) for more than 15 years [47]. A possible effect of *O. volvulus* infection on epilepsy would not be expected at the resulting low level of endemicity. It was mentioned that the mean microfilarial density in the 2040 persons aged ≥15 years examined as part of this study was only 7.5 mf per snip [34], which is low in this historical onchocerciasis focus. Similarly, the study from Mali [31] was conducted in an area which had benefitted from 11 rounds of annual distributions of ivermectin, with drug coverage as high as 66.8% in 1997 while the study was conducted in 1998 [42], plus ground vector control activities during four years [47]. However, if either one of these or all three studies were excluded in the calculation of the combined OR, still a significant result was found for the remaining studies (pooled OR = 2.41 (95%CI: 1.55–3.76), p<0.001; Cochrane's Q test: p = 0.016; I^2^ = 59.4%).

To overcome the apparent weakness of design of some studies, we performed a second step of analysis restricted to those studies giving evidence of control for age, residence and sex as confounding factors of major relevance [20], [29]–[31], [39]. When an analysis was made of the qualitative skin snip data of these four studies, all found an OR above 1 indicating a trend for a positive correlation, but the clearly significant result found for all studies was not confirmed. This may be explained with the limited sample of 367 PWE and 624 PWOE included in the restricted analysis. Alternatively, the highly significant result of the non-restricted analysis might be due to bias from those studies achieving limited control. Actually, unless larger samples are examined with an appropriate protocol, it seems that the qualitative investigation of mf in the skin is of limited use in analyzing the question of an association between onchocerciasis and epilepsy. The first reason is that in highly endemic areas where almost all inhabitants will be infected with *O. volvulus*, such as that studied by Boussinesq et al. in Cameroon [29], the difference in prevalence of infection between cases and controls will be small. On the other hand, in areas of low endemicity a higher fraction of patients with epilepsy due to other causes will be found and this will diminish the strength of a possible association.

In savanna regions, the prevalence of onchocercal eye disease is known to increase exponentially with that of onchocerciasis in a similar way to that observed for epilepsy in *O. volvulus* endemic areas [16], [27]. Eye manifestations are also known to be closely connected with high intensity of infection [3], [27] and it should appear plausible if this is also found with epilepsy. So far, the immediate relation between epilepsy and intensity of infection with *O. volvulus* has been adequately investigated at only one occasion [29]. This study found a significantly higher mf count in dermal biopsies of patients with epilepsy if compared to pair-matched controls without epilepsy when examined prior to ivermectin treatment. Another study reported quantitative results from an area where annual mass treatment had been implemented prior to the investigation [30]. However, after five treatment rounds in this area of the CAR, the strong microfilaricidal effect of ivermectin had, as expected, considerably reduced the microfilarial densities in the population [48]. This effect has probably levelled out a possible difference of infection intensity between PWE and PWOE, which would explain why no significant result was found.

In addition to skin biopsies, two of the studies considered to achieve sufficient control for age, residence and sex also communicated results of nodule palpation [20], [39]. When the results of both these studies were looked at together, a significant result was found for presence of palpable nodules and for the total number of nodules found in the examined sample. As has been mentioned, the sensitivity of nodule palpation is low in patients harbouring only one or few nodules because these may not be accessible. However, the probability that at least one nodule will be palpable increases with nodule load, and it has been demonstrated that, when taking skin snip diagnosis as reference, the sensitivity of nodule palpation exceeds 80% for patients with a total nodule load of 5 or more [7]. Because of the close correlation between nodule load and infection intensity [6], [9], in an onchocerciasis endemic area persons with one or more palpable nodules will be representing the fraction of the population with more intense infection if compared to those persons without a palpable nodule. Therefore, the significant result found for nodule palpation in the present analysis supports the assumption that infection intensity plays a role in the induction of epilepsy in *O. volvulus* endemic areas.

The observation of an unexplained clustering of epilepsy in an endemic focus has been the starting point of most investigations on a possible association between onchocerciasis and epilepsy. As far as detailed information on age-specific epilepsy incidence and prevalence is available from onchocerciasis endemic areas, a consistent distribution has been found with a peak incidence between 10 and 15 years [38], [40], [41], [49], [50], and a peak prevalence in adolescents and young adults [38], [41], [50], [51]. In contrast, studies on epilepsy prevalence from African areas not endemic for onchocerciasis reported minor differences across age groups [52] or highest rates in those younger than 10 years [53]. This unusual age distribution of epilepsy cases in onchocerciasis endemic areas is well compatible with the build-up of *O. volvulus* infection in the population.

Two clinical observations indicate a link between onchocerciasis and epilepsy: (1) A so far poorly understood form of growth failure, which had when first described been named “Nakalanga syndrome” [54] has only been observed in *O. volvulus* endemic areas and an overlap with epilepsy has been described [19], [38], [55], [56]. (2) A specific type of epileptic seizure which has been designated as “head nodding seizures” (HN), again associated with stunted growth, has also been found exclusively in onchocerciasis endemic areas [40], [57]–[61]. Recently, a case-control study conducted in South Sudan reported a significantly higher proportion of infection with *O. volvulus* in patients with HN than in controls matched for age and location of residence [62]. However, it was mentioned that many participants examined with this study were internally displaced persons and the actual duration of exposure to onchocerciasis may have been substantially different between cases and controls depending on their individual history of migration. Further studies using carefully selected controls are needed to confirm - or disprove - the intriguing positive association found in this preliminary investigation [62].

A general problem of cross-sectional studies – as were analyzed with the present review – is that they cannot contribute much to our understanding of exact mechanisms of pathogenesis and also the temporal relationship between the condition assumed as exposure and the outcome cannot be easily studied. One approach to find an answer to the crucial question whether infection with *O. volvulus* is factually preceding the onset of epileptic seizures would be to longitudinally investigate *O. volvulus* infection status of newly incident epilepsy cases and appropriate controls. The so far only prospective assessment of epilepsy incidence data from an onchocerciasis endemic area was carried out on the occasion of an epilepsy treatment programme with repetitive follow up visits [49], [63] in western Uganda. Although with this programme, being based on limited means and having patient care as its focus, individual onchocerciasis infection status was not assessed, this could be done with appropriate and simple protocols in the setting of health facilities or humanitarian programmes engaged in treating epilepsy patients in endemic areas.

Numerous possible infective causes of epilepsy have been described in sub-Saharan Africa [64] and in particular cysticercosis [22] and malaria [65] are considered to be established etiologies. It might be conceivable that epilepsy in the onchocerciasis endemic areas is factually due to some of these factors but an association between the two entities is erroneously found because PWE for various reasons may be more susceptible to *O. volvulus* infection than PWOE. In this case it should be expected that other causes or etiologic factors for epilepsy can be found in patients living in onchocerciasis endemic areas. For instance, it was suggested that neurocysticercosis could be the cause of epileptic seizures in patients living in areas co-endemic for onchocerciasis and cysticercosis [66]. However, although it may happen in some individual patients that nodules due to sub-cutaneous cysticercosis (SCC) are mistaken as *O. volvulus* nodules, this is not expected to play a major role in African endemic areas. The prevalence of SCC there is found to be very low and nodules are usually located on the upper limbs and on the head [67], [68], whereas, at least in Africa, onchocercal nodules are usually localized on the lower part of the body. When the evidence on the inter-relation between onchocerciasis, cysticercosis and epilepsy was reviewed in more depth, the above mentioned presumption was not confirmed [69]. In two larger case series searching for etiologies for epilepsy in patients living in onchocerciasis endemic areas in Tanzania (n = 196) [36], and Uganda (n = 91) [58] no evidence was found for malaria or other infections of the central nervous system as a possible cause. When other suspected pathogens such as infection with arboviruses [70], [71] or *Paragonimus* sp. [72], [29] were investigated, also no connection with epilepsy was found. Thus, as long as no prove is demonstrated of one or several alternative causative factors in PWE to explain the extremely elevated epilepsy incidence [49] and prevalence [16], [51] found in onchocerciasis endemic areas, *O. volvulus* will remain the first suspect.

The pathomechanism by which *O. volvulus* could damage the brain and lead to epileptic seizures is not clarified. *O. volvulus* mf were sporadically revealed in the cerebrospinal (CSF) fluid of patients before and, to an even greater extent, following treatment with diethylcarbamazine [73]–[75], but the examined patients were not affected by epilepsy. When CSF was analysed in patients with epilepsy living in a Tanzanian onchocerciasis endemic area, no mf were found even in patients with documented skin infestation of *O. volvulus* [36], [60]. However, as the activities of the African Programme for Onchocerciasis Control (APOC) started in the Mahenge focus in 1997, i.e. 8 years before the study [37], most of these patients had probably received microfilaricidal treatment with ivermectin prior to their examination and this may have removed mf from the CSF [76]. Magnetic resonance imaging in these patients revealed a number of non-specific pathologic changes which were slightly more frequent in patients with proof of dermal mf [60]. Another route of entry of *O. volvulus* mf into the brain might be the optic nerve, and indeed the parasite has been found in this location on several occasions [77]–[81]. Apart from a direct involvement of one of the developmental stages of *O. volvulus* it has been suggested that an immunological response of the human host, and possibly auto-immune mechanisms such as those found in the development of onchocercal chorioretinitis, may be involved in the pathogenesis of epilepsy [36], [82]–[84]. In view of the scarcity of investigational facilities and neurological expertise in the endemic areas [85], studies on the pathophysiology of onchocerciasis-related epilepsy will remain difficult.

We found a significant association between epilepsy and a positive infection status with *O. volvulus* when all studies identified with an extensive literature search were analysed in combination. This relationship was less pronounced when the analysis was restricted to those studies giving evidence of sufficient control for age, residence and sex. When within this restricted sample indicators giving information on infection intensity, namely nodule palpation and microfilarial density in untreated patients, were taken into consideration, again a significant result was found. Although the overall data base is still of limited size, in their overall constellation these findings corroborate the hypothesis that epilepsy in endemic areas is caused by a disease process induced by infection with *O. volvulus*, especially in patients with severe infection. As already suggested by Balanzario in 1942 [11], infection with *O. volvulus* could be a necessary but not sufficient factor leading to onchocerciasis-associated epilepsy. A better understanding of the dimension and the nature of onchocercal brain disease is needed and will contribute to motivate sustained efforts aiming at the reduction of the disease burden and finally elimination of onchocerciasis.

## Supporting Information

Diagram S1 PRISMA flow diagram.
(PDF)

Checklist S1 PRISMA checklist.
(DOC)

Figure S1 Meta-analysis.
(TIF)
