# Supplementary material for: Case-control Studies on the Relationship between Onchocerciasis and Epilepsy: Systematic Review and Meta-analysis
Source: PLoS Negl Trop Dis. 2013 Mar 28;7(3):e2147. doi: 10.1371/journal.pntd.0002147 (PMC3610636; doi:10.1371/journal.pntd.0002147)

Diagram S1: PRISMA flow diagram

according to PRISMA guidelines (Moher D, Liberati A, Tetzlaff J, Altman DG, The PRISMA Group (2009) Preferred Reporting Items for Systematic Reviews and Meta-Analysis: The PRISMA Statement. PLoS Med 6: e1000097)

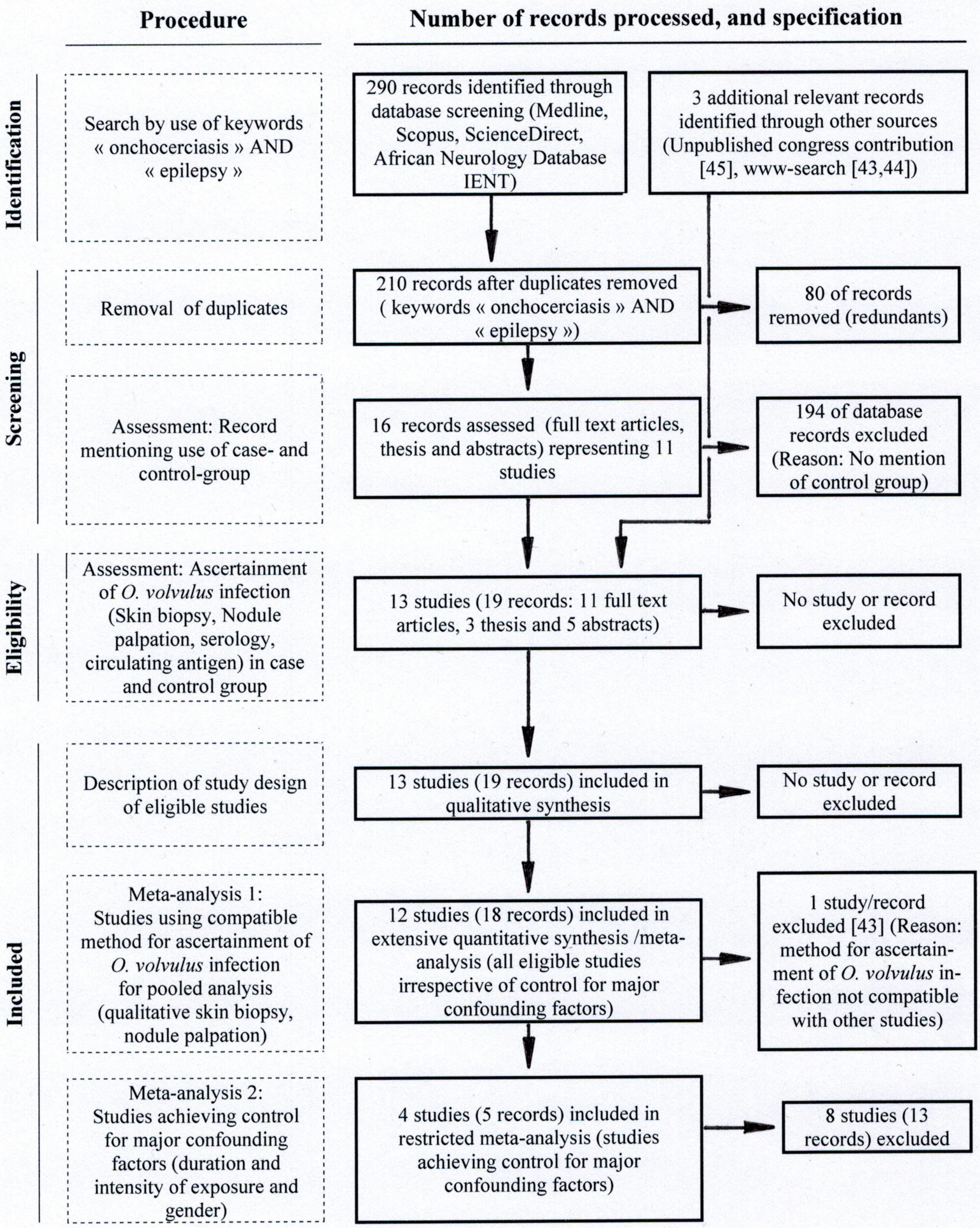

Supplement: Diagram S1 — PRISMA flow diagram. (PDF) [file pntd.0002147.s001.pdf]
