# Supplementary material for: Case-control Studies on the Relationship between Onchocerciasis and Epilepsy: Systematic Review and Meta-analysis
Source: PLoS Negl Trop Dis. 2013 Mar 28;7(3):e2147. doi: 10.1371/journal.pntd.0002147 (PMC3610636; doi:10.1371/journal.pntd.0002147)
Supplement: Checklist S1 — PRISMA checklist. (DOC) [file pntd.0002147.s002.doc]

**Checklist S1:** PRISMA checklist

according to PRISMA guidelines (Moher D, Liberati A, Tetzlaff J, Altman DG, The PRISMA Group (2009) Preferred Reporting Items for Systematic Reviews and Meta-Analysis: The PRISMA Statement. PLoS Med 6: e1000097)

| Section/topic | # | Checklist item | | | Reported on page # |
| --- | --- | --- | --- | --- | --- |
| **TITLE** | | | | | |
| Title | 1 | Identify the report as a systematic review, meta-analysis, or both.  **Both** | | | Page 1 [title page],  Title |
| **ABSTRACT** | | | | | |
| Structured summary | 2 | Provide a structured summary including, as applicable: background; objectives; data sources; study eligibility criteria, participants, and interventions; study appraisal and synthesis methods; results; limitations; conclusions and implications of key findings; systematic review registration number. | | | Page 2, Summary |
| **INTRODUCTION** | | | | | |
| Rationale | 3 | Describe the rationale for the review in the context of what is already known. | | | Page 4, Introduction § 3-4 |
| Objectives | 4 | Provide an explicit statement of questions being addressed with reference to participants, interventions, comparisons, outcomes, and study design (PICOS).   - **Present statement (text, 46 words): “The present article provides a review of all case-control studies investigating the relationship between onchocerciasis and epilepsy carried out to date, with a focus as to whether time and intensity of exposure to *O. volvulus* and gender as the major determinants for infection status were controlled.” Specific information on PICOS is provided in later text sections (Methods, see below).** - **Alternative, more explicit statement (length: 87 words): The present article provides a review of all studies comparing infection status of *O. volvulus* [Intervention] in people with epilepsy (PWE) and without epilepsy (PWOE) [Participants/Comparison]. Details of the design of identified studies are described [Study design]. A meta-analysis is carried out from those studies reporting data allowing for calculation of a pooled odds ratio [Outcome]. In addition, a restricted analysis was carried out on those studies achieving control for time and intensity of exposure to *O. volvulus* and gender as the major determinants for infection status [Control for bias].** | | | Page 5, Introduction § 5 |
| **METHODS** | | | | | |
| Protocol and registration | 5 | Indicate if a review protocol exists, if and where it can be accessed (e.g., Web address), and, if available, provide registration information including registration number.  **See accompanying flowchart and sequence of procedures. Registration intended with publication (if accepted by registry)** | | | Included in flowchart (sequence of procedures) |
| Eligibility criteria | 6 | Specify study characteristics (e.g., PICOS, length of follow-up) and report characteristics (e.g., years considered, language, publication status) used as criteria for eligibility, giving rationale.  **Participants: People with epilepsy (PWE) and without epilepsy (PWOE)**  **Intervention: Assessment of *O. volvulus* infection status (Qualitative data). Assessment of *O. volvulus* infection intensity (Quantitative data; if appropriate)**  **Comparison: Infection status/infection intensity in PWE compared to infection status/infection intensity in PWOE**  **Outcome: (required for eligibility of studies): Data allowing for calculation of odds ratios (qualitative data) or standardized mean differences (quantitative data).**  **Study design: Identification and selection of participants; Assessment of diagnosis of epilepsy and onchocerciasis; Number of participants; Control of confounding factors** | | | Page 5, Methods §1; Page 5-6, Methods §2; Page 7-8, Results §2 ; Tables 1 and 2 |
| Information sources | 7 | Describe all information sources (e.g., databases with dates of coverage, contact with study authors to identify additional studies) in the search and date last searched.  **Databases: MEDLINE, ScienceDirect, Scopus, African Neurology Database of the Institute of Neuroepidemiology and Tropical Neurology of the University of Limoges”.**  **Date of coverage: No time limit for begin of search, last search May 23, 2012 .** | | | Page 5, Methods §1 |
| Search | 8 | Present full electronic search strategy for at least one database, including any limits used, such that it could be repeated.  **MEDLINE: Search “onchocerciasis” AND “epilepsy”, 35 entries retrieved** | | | Page 5, Methods §1 |
| Study selection | 9 | State the process for selecting studies (i.e., screening, eligibility, included in systematic review, and, if applicable, included in the meta-analysis).  **See accompanying flowchart: Sequence of procedures, number and details of processed records.** | | |  |
| Data collection process | 10 | Describe method of data extraction from reports (e.g., piloted forms, independently, in duplicate) and any processes for obtaining and confirming data from investigators. | | | Page 5-6, Methods §1-3 |
| Data items | 11 | List and define all variables for which data were sought (e.g., PICOS, funding sources) and any assumptions and simplifications made.  **Participants: PWE and PWOE**  **Intervention: Assessment of *O. volvulus* infection status**   1. **Qualitative detection of mf of *O. volvulus* in the skin** 2. **Quantitative detection of mf of *O. volvulus* in the skin** 3. **Presence of subcutaneous nodules detected by palpation** 4. **Number of nodules detected by palpation**   **Comparison: Infection status/infection intensity in PWE compared to infection status/infection intensity in PWOE**  **Outcome:**   1. **odds ratio (qualitative data)** 2. **standardized mean difference (quantitative data)**   **Study design:**   1. **Identification and selection of participants** 2. **Assessment of diagnosis of epilepsy and onchocerciasis** 3. **Number of participants** 4. **Control of confounding factors** | | | Page 5-6, Methods §1-2; Page 7-8, Results §2 ; Tables 1 and 2 |
| Risk of bias in individual studies | 12 | Describe methods used for assessing risk of bias of individual studies (including specification of whether this was done at the study or outcome level), and how this information is to be used in any data synthesis.   1. **Rationale for defining major confounding factors (i: Intensity of exposure to *O. volvulus* infection, ii: Time/Duration of exposure, iii: Gender)** 2. **Definition of criteria for control of confounding factors.** 3. **Assessment of predefined criteria in eligible studies (Study level).** 4. **Restricted analysis of studies fulfilling pre-defined criteria (Outcome level).** | | | Page 3, Introduction §1;  Page 5-6, Methods §2; Page 7-8, Methods §2; Table 1; Table 2 |
| Summary measures | 13 | State the principal summary measures (e.g., risk ratio, difference in means).  **1. Pooled odds ratio (for qualitative indicators)**  **2. Pooled standardized mean difference (for quantitative indicators)** | | | Page 6, Methods §1 and 2 |
| Synthesis of results | 14 | Describe the methods of handling data and combining results of studies, if done, including measures of consistency (e.g., I^2^) for each meta-analysis.  **Pooled ORs and 95% CI were calculated for studies presenting qualitative indicators of onchocerciasis infection whereas pooled standardized mean differences (SMD) and Cohen’s d statistics were computed for studies presenting quantitative assessment of onchocerciasis infection.** **Pooled ORs were estimated using random-effects models (DerSimonian-Laird method). Heterogeneity of studies was assessed using Chi-squared tests (Cochran's Q test) and I^2^ values.** | | | Page 6, Methods §1 and 2 |
| Risk of bias across studies | 15 | Specify any assessment of risk of bias that may affect the cumulative evidence (e.g., publication bias, selective reporting within studies).  **Risk of bias has been minimized during the study selection phase (comprehensive search of formally published and grey records in medical databases AND commercial search engines; exclusion of inappropriate surveys). Pooled OR re-assessed after exclusion of three possibly biased studies [ref. 18;30;32-34] (addressed in the context of the discussion).** | | | Page 5, Methods §1; See also flowchart;  Pages 9-10, Discussion §1 |
| Additional analyses | 16 | Describe methods of additional analyses (e.g., sensitivity or subgroup analyses, meta-regression), if done, indicating which were pre-specified.  **Subgroup analysis; analysis restricted to studies achieving control for pre-specified confounding factors.** | | | Page 8, Results §2-4, Table 3 |
| RESULTS | | | | | |
| Study selection | 17 | Give numbers of studies screened, assessed for eligibility, and included in the review, with reasons for exclusions at each stage, ideally with a flow diagram. | | See accompanying flow chart | |
| Study characteristics | 18 | For each study, present characteristics for which data were extracted (e.g., study size, PICOS, follow-up period) and provide the citations. | | Presented in detail in Tables 1 and 2 | |
| Risk of bias within studies | 19 | Present data on risk of bias of each study and, if available, any outcome-level assessment (see Item 12). | | Table 2 (risk of bias),  Table 3, restricted analysis (outcome level) | |
| Results of individual studies | 20 | For all outcomes considered (benefits or harms), present, for each study: (a) simple summary data for each intervention group and (b) effect estimates and confidence intervals, ideally with a forest plot. | | Presented in Table 2 and 3. No forest plot provided (redundant information with Tables) | |
| Synthesis of results | 21 | Present results of each meta-analysis done, including confidence intervals and measures of consistency. | | Page 7-8;  See Tables 2 and 3 | |
| Risk of bias across studies | 22 | Present results of any assessment of risk of bias across studies (see Item 15).  **Heterogeneity of studies was assessed using Chi-squared tests (Cochran's Q test) and I^2^.** | | Page 8-9; Results | |
| Additional analysis | 23 | Give results of additional analyses, if done (e.g., sensitivity or subgroup analyses, meta-regression [see Item 16]).  **Analysis of subgroup (restricted analysis) of studies achieving control for pre-specified confounding factors.**  **In the footnotes for Table 2, we made some assumptions regarding the onchocerciasis status of 6 control individuals with missing data and calculated ORs according to these different assumptions.** | | Page 8-9; Results §2-4,  Tables 2 and 3, footnotes | |
| DISCUSSION | | | | | |
| Summary of evidence | 24 | Summarize the main findings including the strength of evidence for each main outcome; consider their relevance to key groups (e.g., health care providers, users, and policy makers). | Page 9-11, §1, sentence 1-2; Page 10, §2, sentence 1-3; Page 11-12, §3, sentence 3-6, Page 11, §4, sentence 1-2 ; Page 13, §7 (final paragraph) | | |
| Limitations | 25 | Discuss limitations at study and outcome level (e.g., risk of bias), and at review level (e.g., incomplete retrieval of identified research, reporting bias). | Page 9-11, §1, sentence 3 - end of §; §2, sentence 4 – end of §; §3, sentence 6 – end of §; §4, sentence 3 – end of §. | | |
| Conclusions | 26 | Provide a general interpretation of the results in the context of other evidence, and implications for future research. | Page 11 – 13, §5 - §6, §7 (final) | | |
| FUNDING | | | | | |
| Funding | 27 | Describe sources of funding for the systematic review and other support (e.g., supply of data); role of funders for the systematic review. | No funding was received for the review. Support of researchers for supply of additional information is indicated in the acknowledgement section. | | |
