# Supplementary figures and images for: Case-control Studies on the Relationship between Onchocerciasis and Epilepsy: Systematic Review and Meta-analysis
Source: PLoS Negl Trop Dis. 2013 Mar 28;7(3):e2147. doi: 10.1371/journal.pntd.0002147 (PMC3610636; doi:10.1371/journal.pntd.0002147)

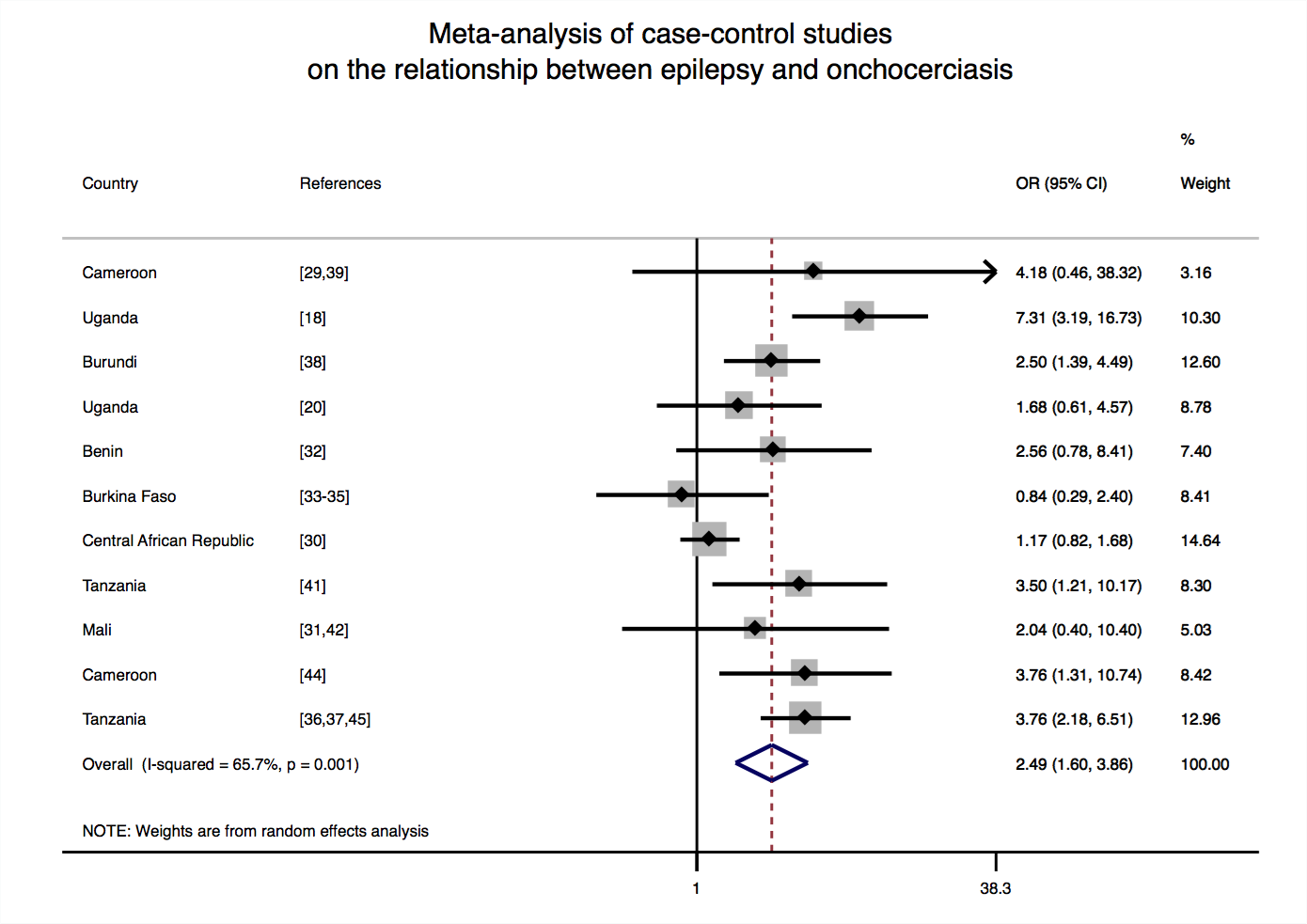

Supplement: Figure S1 — Meta-analysis. (TIF) [file pntd.0002147.s003.tif]
